# Differences in the regeneration traits of *Potamogeton crispus* turions from macrophyte- and phytoplankton-dominated lakes

**DOI:** 10.1038/srep12907

**Published:** 2015-08-06

**Authors:** Dong Xie, Hengjie Zhou, Hong Zhu, Haiting Ji, Ning Li, Shuqing An

**Affiliations:** 1 Co-Innovation Center for Sustainable Forestry in Southern China, College of Biology and the Environment, Nanjing Forestry University, Nanjing 210037, P. R. China; 2 School of Life Science and Institute of Wetland Ecology, Nanjing University, Nanjing 210093, P. R. China; 3 Nanjing University Ecology Research Institute of Changshu (NJUecoRICH), Changshu 215500, Jiangsu, P. R. China

## Abstract

*Potamogeton crispus* is widely used in submerged macrophyte restoration in China. Turions are an important means of reproduction in this species. To compare the regeneration abilities of *P. crispus* turions in macrophyte- and phytoplankton-dominated lakes, we collected *P. crispus* turions from a macrophyte-dominated lake (Liangzi Lake) and a phytoplankton-dominated lake (Taihu Lake). Both lakes are important lakes in the middle and lower reaches of the Yangtze River in China. Our field survey revealed that the turions from the phytoplankton-dominated lake had smaller sizes and higher concentrations of total nitrogen (TN) and total phosphorus (TP) than did those from the macrophyte-dominated lake. Rapid sprouting of the turions from the phytoplankton-dominated lake in 32 days was observed under experimental conditions, although the sprout sizes (heights and biomass) were smaller than those from the macrophyte-dominated lake. Compared with sprouted turions from macrophyte-dominated lake, the sprouted turions from the phytoplankton-dominated lake accumulated higher soluble sugar (SS) but lower starch and free amino acid (FAA) concentrations. A 12-day interval sprout removal treatment significantly stimulated the re-sprouting of turions from both lakes, but scale-leaf-removal treatments had no effect. This study provides evidence that the regeneration strategies of *P. crispus* turions differ in macrophyte- and phytoplankton-dominated lakes.

Successful regeneration from propagules is a crucial determinant that regulates the temporal and spatial dynamics of plant communities and influences the management of habitat conservation and restoration in aquatic ecosystems^1^,^2^,^3^,^4^. Submerged macrophytes can facilitate the clear-water conditions in shallow lakes^5^,^6^. Many submerged macrophytes produce aboveground vegetative organs (or asexual propagules) that detach from their mother plants at the end of each growing season and sprout in the next season^7^,^8^. Therefore, propagule regeneration capacity is an important issue in population ecology^9^.

Individual plants tend to exhibit a trade-off between current growth and stored resources for future survival and recovery^10^. For plants that grow under stressful conditions (e.g., low resource availability or physical damage), high proportions of resources are allocated to the storage organs (e.g., stems or asexual propagules) for future plant regeneration^7^,^11^. Large propagules in nature represent a relatively large investment in nutrient and carbohydrate storage, which can facilitate a robust re-sprouting response when these propagules are activated^12^,^13^,^14^. For instance, tuber sizes mediated the local adaptation of the submerged macrophyte *Potamogeton pectinatus*^15^. Several other studies in aquatic plants also revealed that the plant regeneration capacities were related to the size of the storage organs^16^,^17^,^18^. Moreover, in addition to the storage organ sizes, the quality and quantity of resources stored in their storage organs may influence the regeneration process^10^. Surprisingly, although extensive literature has focused on plant regeneration, few papers have considered the possible effects of environmental stresses.

Eutrophication is a worldwide process that affects most aquatic ecosystems and their associated services^19^,^20^. In these eutrophic aquatic systems, plant regeneration and dispersal rely strongly on vegetative means^2^,^21^,^22^,^23^, and eutrophication may negatively affect the tolerance of plant species by, for example, reducing biomass or decreasing regeneration efficiency. Recent studies have demonstrated that, under high-nutrient experimental conditions (both sediment and water column), some submerged macrophytes tend to exhibit a reduced size of asexual propagules [e.g.^24^,^25^,]. Moreover, other factors associated with eutrophication (e.g., anoxia, low light and sediment nutrient conditions) have a major influence on propagation and the propagule regeneration capacity of submerged macrophytes^16^,^26^,^27^, although every asexual propagule theoretically has the capacity to initiate a new shoot to sustain the population^28^. Therefore, determining the effect of eutrophication on plant life history traits related to growth, storage and propagule regeneration capacity is a key component of elucidating patterns of biodiversity losses in aquatic ecosystems facing eutrophication. Such a comparison will also facilitate the formulation of effective management schemes to prevent the further degradation of biodiversity in highly threatened areas at the global scale.

Turions of submerged macrophytes are tough, sturdy organs that are formed by specialized shoot apices^29^. The main function of the turion is storage to support the growth of new roots/shoots in the next growing season^29^,^30^. For instance, turions of submerged macrophytes store large amounts of starch (9–70% dry weight [DW]), free sugars (7–14% DW)^31^,^32^,^33^,^34^,^35^ and mineral nutrients (N and P)^30^. However, in nature, the sprouting capacity of these propagules may differ significantly because of their specific characteristics and different environmental conditions^14^,^15^. For instance, a previous study found that most *Potamogeton crispus* turions, without experiencing sprout removal, produced one sprouting shoot, and those experiencing shoot removal produced a second or even third re-sprouting shoots^36^. For both turion sprouting and re-sprouting process, a complex carbohydrates metabolism is involved^37^. Moreover, turions can disperse by water flow and recolonize new habitats^38^. During the dispersal phase, sprout/shoot breakage is normal; therefore, the turion re-sprouting capacity is important for the survival and further dispersal of submerged macrophytes. However, the effects of asexual propagule properties on plant regeneration have largely been overlooked^39^.

*Potamogeton crispus* L. is a submerged macrophyte that is widely distributed throughout China and is often used in ecological restoration for eutrophic sites^7^,^25^. From late spring to early summer, turions of *P. crispus* are formed when the temperature is higher than 20°C and/or the photoperiod is longer than 12 h^26^. The newly formed turions are hard, green, bur-like dormant buds on the stem with crowded, small holly-like leaves (scale leaves). In natural water bodies, the turions usually remain dormant for several weeks to months, but they break dormancy under certain experimental temperature and/or light conditions (e.g., temperatures under 23 °C)^26^,^37^,^40^. Our previous studies have suggested that nutrients regulate scale leaf morphology, which determines the turion sizes and, in turn, influences the turion sprouting process^17^,^41^. However, no study has established direct links between lake nutrients status (e.g., macrophyte-dominated lake versus phytoplankton-dominated lake) and the turion regeneration traits of submerged macrophytes. Moreover, few studies have investigated the effect of internal turion features (e.g., scale leaf) on the sprouting/re-sprouting capacities and plant growth of *P. crispus* turions. In the present study, we compared *P. crispus* turions and their sprouting/re-sprouting capacities between a macrophyte-dominated lake and a phytoplankton-dominated lake. Specifically, we investigated the following topics: 1) whether turions and their sprouting/re-sprouting traits from a phytoplankton-dominated lake differ from those from a macrophyte-dominated lake; 2) whether different turions (specifically the stem and scale leaf) play different roles in the sprouting/re-sprouting process.

## Materials and Methods

### Collection site

In late June 2012, we collected turions of *P. crispus* from 6 field sites (3 sites per lake) in two lakes: Liangzi Lake in Hubei Province (30°05′-30°18′N, 114°21′-114°39′E) and Taihu Lake in Jiangsu Province (30°56′-31°33′N, 119°54′-120°36′E) (Table 1). These lakes are both important lakes in the middle and lower reaches of the Yangtze River. Liangzi Lake is a macrophyte-dominated lake (Chlorophyll-*a* maximum concentration 40.9 μg L^−1^), and Taihu Lake is a phytoplankton-dominated lake (Chlorophyll-*a* maximum concentration 148.3 μg L^−1^)^42^,^43^. At each site, 600 to 800 turions were collected by using a Peterson dredge (1/16 m^2^). Upon collection, the turions were kept in plastic boxes filled with lake water and transported to the laboratory within 48 h. Then, the turions were washed with tap water to remove the mud and epiphytes and then separated into the Liangzi Lake and Taihu Lake groups before they were subjected to either sprouting experiment or chemical analysis.

Physical characteristics (i.e., water depth, pH, dissolved oxygen and turbidity) were measured with a handheld multi-parameter meter (Proplus, YSI, USA) at each collection site from 10:00 to 15:00. At each collection site, three water samples at a depth of 50 cm were firstly filtered by GF/F filter, and then were used for water chemical characteristics measurement using a ion chromatography system (ICS-1000, Dionex, Sunnyvale, CA, USA) to determine the 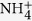, 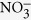, and 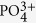 within 48 h. Three sediment samples were also collected and transported to the laboratory. The sediment samples were then air dried and ground for total nitrogen (TN), total phosphorus (TP) and organic matter analysis.

### Experimental design

#### Sprouting and re-sprouting trait measurement

All experiments were conducted at the Institute of Wetland Ecology, Nanjing University, Nanjing, China, and lasted for 32 days. To compare the turion sprouting capacities between the two lakes, 15 turions from one collection site, which had similar sizes (fresh weight: Taihu Lake: 0.72–0.88 g; Liangzi Lake: 0.97–1.45 g), were placed into 3 plastic bowls (5 turions per bowl, diameter 10 cm, height 15 cm). The bowls from all collection sites were filled with tap water (NH_4_-N: <0.025 mg L^−1^; NO_3_-N: 0.85–1.14 mg L^−1^; PO_4_-P: undetectable; pH: 6.8–7.8) and were then randomly placed in a light incubator (light:dark period ratio = 16 h:8 h). There were 3 replications for each collection site; consequently, a total of 90 turions were used in the sprouting experiment. The water in the bowls was changed every 3 days. The light intensity was 110 ± 5 μmol/m^2^·s, and the temperature was 15 ± 1 °C, which is consistent with the optimal sprouting conditions for *P. crispus* turions in China^26^,^40^.

To compare the turion re-sprouting capacities between the two lakes, another 15 turions from one collection site, which had similar sizes (fresh weight: Taihu Lake: 0.66–0.86 g; Liangzi Lake: 1.05–1.32 g), were also placed into 3 plastic bowls (5 turions per bowl, diameter 10 cm, height 15 cm). These turions were placed in the light incubator with the same sprouting conditions described above. However, the sprouts were removed with scissors in 12-day intervals. There were 3 replications for each collection site; consequently, a total of 90 turions were used. In both the sprouting and re-sprouting experiments, the number of sprouted turions was recorded every 4 days. At the end of the experiment (day 32), the sprouting shoot number, shoot length and shoot biomass were also recorded.

#### Scale-leaf-removal sprouting experiment

To investigate whether turion scale leaves influence sprouting, a scale-leaf-removal sprouting experiment was conducted. The scale leaves of turions were subjected to 3 types of removal: 100% removal, 50% removal or no removal (control). Within each treatment, 5 turions of similar size from the same collection site were placed into a plastic bowl (diameter 10 cm, height 15 cm), which was filled with tap water. Each treatment was repeated 3 times, and, as a result, 54 bowls and 270 turions (fresh weight: Taihu Lake: 0.65–0.92 g; Liangzi Lake: 1.15–1.44 g) were used in this experiment. All bowls were randomly placed into two light incubators (light:dark period ratio: 16 h:8 h; the light intensity was 110 ± 5 μmol m^−2^·s^−1^, and the temperature was 15 ± 1 °C). The water in the bowls was renewed every 3 days. The sprouted turions were counted and recorded every 4 days. During the sprouting period, new shoots (>0.5 cm) from the sprouted turions were removed by scissors every 12 days to study the re-sprouting capacities.

### Chemical analysis

Upon collection, 10 turion samples from each collection site were dried at 80 °C for 72 h and then ground to a powder for TN and TP analysis. The TN concentrations of turion and sediment samples were analyzed using a Euro EA3000 elemental analyzer (EuroVector^®^ Instruments and Software, Milan, Italy). The turion and sediment samples were digested with the HCLO_4_-H_2_SO_4_ method^44^ and analyzed using an IL-500P phosphorus analyzer (Hach^®^ Company, Loveland, Colorado, USA) for the TP concentration.

To study the traits of the chemical composition of turions from the two lakes, we measured the soluble sugar (SS), starch and free amino acid (FAA) levels in the turions. In both the Taihu and Liangzi Lake groups, 30 non-sprouted turions and 30 sprouted turions (5 turions from each collection site) were randomly chosen for the measurement after the 32-day sprouting experiment. The measurement of SS and starch was performed using the anthrone method^45^, and the FAA was measured using the ninhydrin colorimetric method^46^.

### Statistical analyses

The turion sprouting and re-sprouting rates were analyzed using repeated measures analysis of variance (ANOVA), with the turion group (Liangzi group versus Taihu group) and/or scale-leaf-removal level as the fixed factors. The differences among collection sites were treated as random effects, and there were no significant effects (P > 0.05). Therefore, the sites were removed from the analyses. The differences in the internal characteristics (e.g., SS, starch and FAA concentrations) between the turions from two lakes in different sprouting stages were analyzed using two-way ANOVA with a Duncan correction, with the turion group (Liangzi group versus Taihu group) and turion sprouting status (non-sprouted versus sprouted) as fixed factors. If the experimental data did not meet homogeneity of variance or normal distribution of residuals, the data were first transformed using a log(x) function and then analyzed. All of the data were analyzed using the SPSS 19.0 program (SPSS, Chicago, IL, USA).

## Results

### Turion sprouting

**Figure 1:**
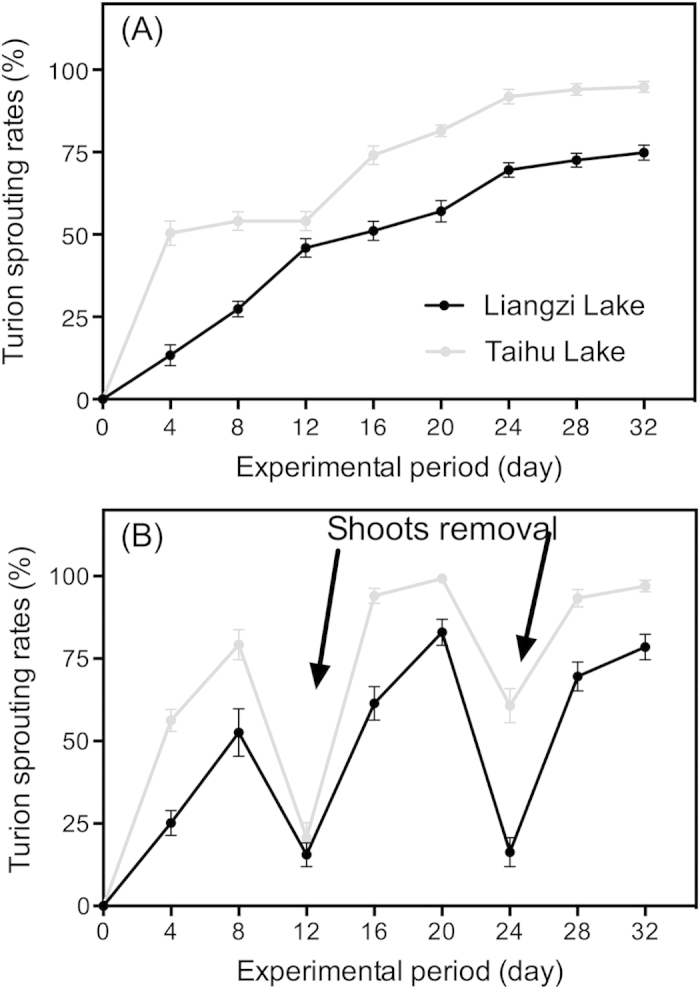
*P. crispus* turion sprouting rates (**A**) and re-sprouting capacities (**B**) between the macrophyte- and phytoplankton-dominated lakes. To test the turion re-sprouting capacities, the sprouts were removed in 12-day intervals. The 32-day sprouting experiments were conducted in light incubators (light intensity: 110 ± 5 μmol m^−2^s^−1^ and air temperature: 15 ± 1 °C). The data are presented as the means ± SE (n = 3).

**Figure 2:**
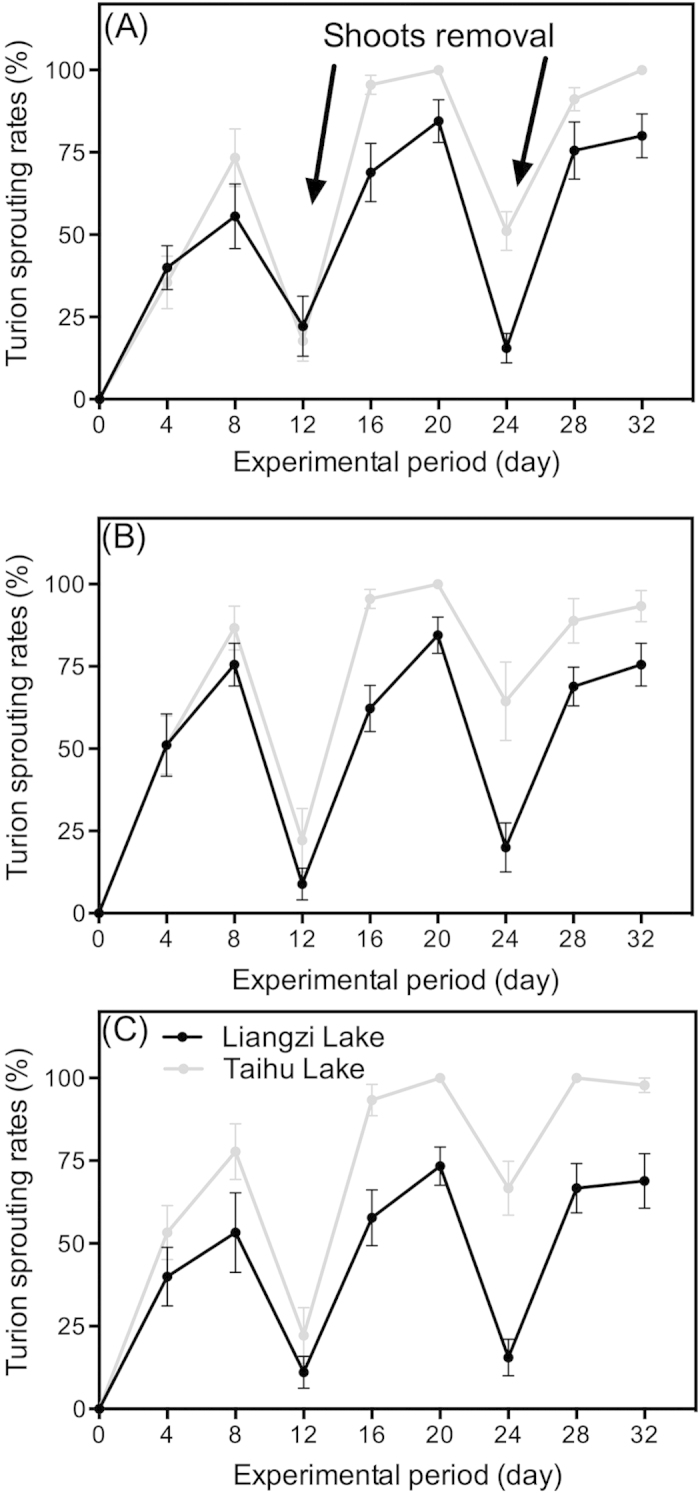
*P. crispus* turion sprouting rates after 100% scale-leaf removal (**A**), 50% scale-leaf removal (**B**) and control (**C**). The 32-day sprouting experiments were conducted in light incubators (light intensity: 110 ± 5 μmol m^−2^s^−1^ and air temperature: 15 ± 1 °C). The data are presented as the means ± SE (n = 3).

During the experimental period, the sprouting ratios of turions from Taihu Lake were higher than those of turions from Liangzi Lake during the sprouting period (Fig. 1A, Table 2). For the turions experiencing sprout removal treatment, turions from the Taihu group also had a higher sprouting ratio (Fig. 1B, Table 2). In both treatments, the sprouting of turions from Taihu Lake was significantly quicker than that of the Liangzi Lake turions. Sprout removal stimulated the sprouting of turions, and over 60% (Liangzi Lake) and 90% (Taihu Lake) of turions sprouted within 8 days, respectively (Fig. 1). Moreover, scale-leaf removal had no significant effect on the turion sprouting ratio (Table 2). For all three levels of scale-leaf removal, turions from Taihu Lake had significantly higher sprouting ratios than those from Liangzi Lake (Fig. 2, Table 2).

**Figure 3:**
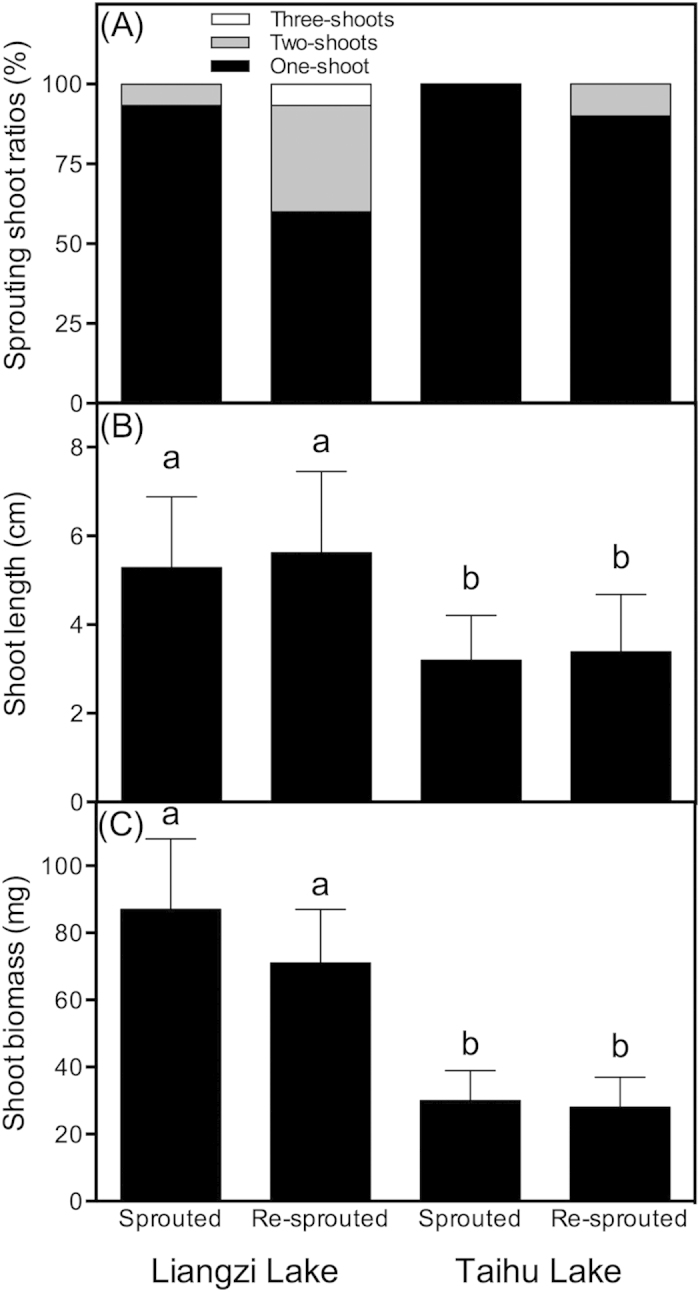
Sprouting shoot ratios (**A**), shoot length (**B**) and shoot biomass (**C**) of macrophyte- and phytoplankton-dominated lakes with sprouted and re-sprouted (shoot removal) turions. (**B,C**) data are presented as the means ± SE (n = 3). Bars sharing the different letters indicate significant differences among the treatments (*P *< 0.05, two-way ANOVA with a Duncan correction). Data were transformed using the log(x) function.

In the sprouting treatment, most of the sprouted turions produced only one sprout. However, after the sprouts were removed, turions from both lakes exhibited a significantly higher second shoot ratio, and some turions from Liangzi Lake even produced a third sprout. Moreover, shoot removal had no significant effect on the shoot length or shoot biomass for turions from both lakes (Fig. 3). In both treatments, turions from Liangzi Lake had higher shoot lengths and biomass accumulations than those from Taihu Lake.

### Chemical concentrations

The turions from Taihu Lake accumulated more TN and TP than those from Liangzi Lake. In turions from Taihu Lake, the TN and TP concentrations were 1.40 ± 0.04% and 2111.058 ± 63.611 μg g^−1^, respectively; whereas, in turions from Liangzi Lake, the TN and TP concentrations were 1.18 ± 0.04% and 1641.969 ± 68.073 μg g^−1^, respectively (mean ± SE, n = 10).

**Figure 4:**
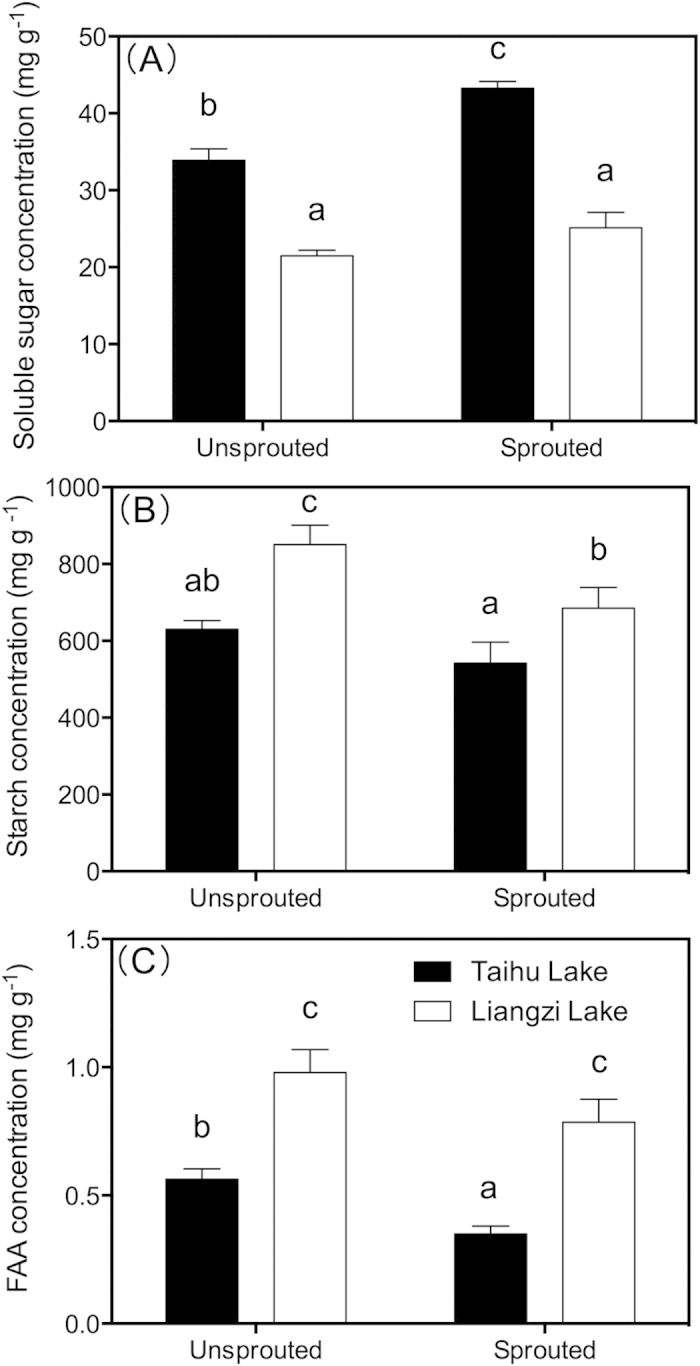
The SS concentration (**A**), starch concentration (**B**) and FAA concentration (**C**) in unsprouted and sprouted turions between the macrophyte- and phytoplankton-dominated lakes. The data are the presented as the means ± SE (n = 5). The bars sharing different letters indicate significant differences among the treatments (*P *< 0.05, two-way ANOVA with a Duncan correction). Data were transformed using the log(x) function.

For the non-sprouted turions, the SS concentrations in turion from Taihu Lake were significantly higher than in turions from Liangzi Lake. Similarly, for the sprouted turions, turions from Taihu Lake had significantly higher SS concentrations than those from Liangzi Lake. For the turions from Taihu Lake, the SS concentrations in sprouted turions were significantly higher than those in non-sprouted turions. However, for the turions from Liangzi Lake, similar SS concentrations were observed between sprouted and non-sprouted turions (Fig. 4A).

For the non-sprouted turions, the starch concentrations in turions from Liangzi Lake were significantly higher than in turions from Taihu Lake. Similarly, for the sprouted turions, turions from Liangzi Lake had significantly higher starch concentrations than those from Taihu Lake. For the turions from both lakes, the starch concentrations in sprouted turions were lower than those in the non-sprouted turions, but only the turions from Liangzi Lake exhibited a significant reduction (Fig. 4B).

For the non-sprouted turions, the FAA concentrations in turions from Liangzi Lake were higher than those from Taihu Lake. For the sprouted turions, no significant difference in the FAA concentration was observed between turions from Liangzi Lake. The FAA concentrations of turions from Taihu Lake were significantly reduced in the sprouted turions (Fig. 4C).

## Discussion

Our results revealed that the regeneration strategies of *P. crispus* turions differed between the macrophyte- and phytoplankton-dominated lakes. The field survey showed that the sizes of turions from the phytoplankton-dominated lake (Taihu Lake) (fresh weight from 0.45 to 1.02 g) were smaller than those from the macrophyte-dominated lake (Liangzi Lake) (fresh weight from 0.88 to 2.21 g). Higher concentrations of TN and TP were also observed in turions from phytoplankton-dominated lake than those from the macrophyte-dominated lake. The turions from the phytoplankton-dominated lake sprouted more quickly during a 32-day interval sprouting experiment, whereas the sprouts sizes (heights and biomass) were smaller than those from macrophyte-dominated lake. Moreover, the sprouted turions from phytoplankton-dominated lake accumulated more SS, whereas their starch and FAA concentrations were lower than those from the macrophyte-dominated lake. A 12-day interval sprout removal treatment significantly stimulated the re-sprouting of turions from both lakes, but there was no effect in the scale-leaf-removal treatments.

The turion sizes were different between the macrophyte- and phytoplankton-dominated lakes. This pattern is consistent with recent evidence that *P. crispus* tends to produce small turions under high sediment/water nutrient conditions^7^,^25^. Under low-nutrient availability conditions, plants decrease their investment in newly growing propagules, resulting in a larger turion production compared with plants in high-nutrient availability conditions^7^,^15^. Previous studies also reported that changes in the water/sediment nutrient concentrations (i.e., TN and TP) trigger changes in the internal N-P metabolism in *P. crispus*, which has a direct effect on chlorophyll synthesis and photosynthesis, affecting turion production and reserves (i.e., turion size and TN and TP concentration differences in our field survey)^7^,^25^,^47^. Under natural habitats, relatively smaller propagules may be produced by several disturbance factors, such as waves^24^. Taihu Lake (2250 km^2^) is much larger than Liangzi Lake (304 km^2^). It is possible that large waves in Taihu Lake would cause the early abscission of newly formed turions and reduce the turion formation period, which can reduce the size of turions. Furthermore, the relatively lower water clarity in Taihu Lake, because it is dominated by phytoplankton, may reduce the material and light energy availability, leading to the propagule size reduction^25^,^48^. The effect of light stress from phytoplankton is also thought to be a key factor underlying declines in the submerged macrophyte vegetation in many places worldwide^48^.

The SS and starch concentrations in non-sprouted turions (new collection turions) differed significantly between the two nutrient-level lakes, the increase/reduction of SS/starch concentrations are consistent with previous studies performed under different water phosphorus conditions, also indicating *P. crispus* from phytoplankton-dominated lake experienced high phosphorus stress^25^. In the sprouted turions from both lakes, the SS concentrations in turions were increased, whereas the starch concentrations in turions were reduced comparing with the non-sprouted turions, although this trend is only significant in SS in turions from phytoplankton-dominated lake and in starch in turions from macrophyte-dominated lake. The increased SS concentrations were consistent with the fast sprouting in turions from phytoplankton-dominated lake. Previous studies have indicated that the initial SS concentrations, other than starch concentrations, in propagules were crucial to their sprouting^14^,^17^. The starch reserves do not directly drive the propagule sprouting process, but they must be transformed to soluble sugar for utilization by the plants^49^,^50^. The starch concentration is usually decreased over several days/hours after the activation of α-amylase and starch phosphorylase^51^,^52^. The increased SS concentrations of turions from both lakes in our study were attributed to the starch amylolysis process, which is consistent with a recent study that starch concentrations reduced after turion sprouting^37^. However, the starch concentrations did not differ significantly between non-sprouted and sprouted turions from phytoplankton-dominated lake. This may attribute to that most current-season-formed turions can accumulate starch during a short period (weeks)^37^. However, mechanisms underlying turion sprouting, which involves the metabolism of multiple carbohydrates, is still unclear, and further study is needed. Furthermore, significant differences in turion sprouting and turion SS concentration were observed between the macrophyte- and phytoplankton-dominated lakes, indicating that the SS concentration-regulated turion sprouting may depend on the environment.

Moreover, the FAA concentration in turions from the phytoplankton-dominated lake was significantly lower than that in the turions from the macrophyte-dominated lake. This difference might be ascribed to the reduction in the FAA levels caused by protein synthesis. For instance, previous studies suggested that the FAA concentration of submerged macrophytes were negatively correlated with plant growth/turion sprouting^26^,^53^, which is consistent with our observation that turions from the phytoplankton-dominated lake sprouted faster than turions from the macrophyte-dominated lake in the sprouting experiment. The concentrations of these important materials supported the different relationship between the speed and strength of turions from the phytoplankton-dominated and macrophyte-dominated lakes.

Our results also showed that the TN and TP concentrations were significantly higher in turions from the phytoplankton-dominated lake than turions from the macrophyte-dominated lake. This result is consistent with our previous findings that sediment nutrients enrichment increased the TN and TP accumulation in turions^7^. The high TN and TP concentrations in turions from the phytoplankton-dominated lake would support their fast sprouting and growth because more TN and TP (particularly TP) is allocated to RNA for synthesis proteins that are required for fast growth^54^,^55^. For example, a previous study revealed that the turion sprouting process (starch breakdown and growth of new photosynthetic organ) of *Spirodela polyrhiza* depends on the availability of nitrogen to achieve the necessary energy metabolism and biosynthesis reactions^56^.

For all the treatments in our study, the turions from the phytoplankton-dominated lake sprouted faster than the turions from the macrophyte-dominated lake. However, relatively smaller sprouts (shorter and small biomass) were observed in turions from the phytoplankton-dominated lake. These results suggest the turions from phytoplankton-dominated lake have low regeneration capabilities. Indeed, recent studies in aquatic invasive/exotic species have reported that exotic propagules with leaves are more invasive than those without leaves because the leaves can act as primary carbohydrate producers through photosynthesis^16^,^57^. Our result also revealed that sprout removal stimulated the re-sprouting of turions from both lakes. However, turions from the phytoplankton-dominated lake had a lower re-sprouting rate than those from the macrophyte-dominated lake. Although turions from the phytoplankton-dominated lake tended to re-sprout more quickly in response to sprout removal, more shoots were produced by turions from the macrophyte-dominated lake. These results also indicated that turions from the macrophyte-dominated lake had greater regeneration capacities than the turions from the phytoplankton-dominated lake. This might be an important reason causing submerged macrophyte species losses and ecosystem instability in phytoplankton-dominated lakes^48^,^58^. Moreover, Bakker *et al.*^3^ reviewed the importance of remnant propagule banks on submerged macrophyte returning. Our results also suggested that the restoration of submerged macrophyte in phytoplankton-dominated lakes may difficult (or slow) because of the unfavourable environment (due to water turbidity) and the weaker regeneration capacities of remnant propagules. Therefore, it is imperative to focus on the recruitment phase of submerged macrophytes more closely for restoring submerged macrophyte communities in phytoplankton-dominated lakes.

Turions from both lakes exhibited no significant difference in their response to the three levels of scale-leaf removal, supporting our previous preliminary findings that the main function of the scale leaf of *P. crispus* turions is dispersal (i.e., regulating turion sinking and floating by scale leaf porosity)^17^,^40^. To the best of our knowledge, however, no similar studies on aquatic plants have focused on the mechanism of the break in *P. crispus* turion dormancy (except for^37^). Therefore, future studies should further investigate how environmental conditions regulate the sprouting/dormancy of *P. crispus* turions.

## Conclusion

In phytoplankton-dominated lakes, a lower regeneration efficiency of submerged macrophytes may cause the decline of submerged macrophyte biomass and species loss in response to stress conditions such as low light and anoxia^4^,^48^. In the present study, we investigated the regeneration capacities of *P. crispus* turions between a macrophyte-dominated lake and a phytoplankton-dominated lake. The turions from the latter lake had smaller sizes than those from the former lake. Higher concentrations of TN and TP were stored in turions from the phytoplankton-dominated lake compared with turions from the macrophyte-dominated lake. Moreover, in the 32-day sprouting experiment, the turions from the phytoplankton-dominated lake sprouted more quickly, whereas the sprout sizes (sprout heights and biomass) were smaller than those of the turions from the macrophyte-dominated lake. In the sprouted turions from the macrophyte-dominated lake, higher SS concentrations accumulated; however, the starch and FAA concentrations were reduced, indicating the high levels of metabolites in these turions. The sprout removal, but not scale-leaf removal, significantly stimulated the sprouting of turions from both lakes. This study provides evidence that regeneration strategies of *P. crispus* turions differed between the macrophyte- and phytoplankton-dominated lakes. Our results also offered new insights into the vegetative reproduction studies of the submerged macrophyte population.

## Additional Information

**How to cite this article**: Xie, D. *et al.* Differences in the regeneration traits of *Potamogeton crispus* turions from macrophyte- and phytoplankton-dominated lakes. *Sci. Rep.* **5**, 12907; doi: 10.1038/srep12907 (2015).

## Figures and Tables

**Table 1:** Principal characteristics of the six collection sites from a macrophyte-dominated lake (Liangzi Lake) and a phytoplankton-dominated lake (Taihu Lake).

Collection site	Water depth (m)	Water						Sediment			
		pH	Dissolved oxygen (mg L^−1^)	Turbidity (NTU)	NH_4_-N (mg L^−1^)	NO_3_-N (mg L^−1^)	PO_4_-P (μg L^−1^)	TN (mg g^−1^ DW)	TP (mg g^−1^ DW)	Organic matter (%)	
Liangzi Lake											
30°14′42″N, 114°33′15″E	2.5	7.20	6.39	3.44	0.09–0.17	0.09–0.19	7.50–7.90	1.44–1.88	0.11–0.22	9.57–11.36	
30°16′20″N, 114°35′40″E	2.0	6.88	7.88	3.39	0.12–0.23	0.08–0.22	8.55–8.99	1.77–2.34	0.11–0.21	8.29–9.76	
30°17′02″N, 114°35′30″E	2.3	6.89	8.21	4.05	0.10–0.22	0.27–0.40	9.20–10.23	1.99–2.54	0.16–0.30	12.2–14.34	
Taihu Lake											
30°56′55″N, 120°16′42″E	1.8	7.82	7.95	232.2	0.66–1.23	0.17–0.22	35.33–40.76	2.02–2.54	0.55–0.73	8.72–10.33	
30°56′39″N, 120°19′50″E	1.8	7.95	7.44	178.9	0.65–0.97	0.10–0.14	22.34–26.47	2.23–2.78	0.63–0.76	10.13–12.79	
30°57′33″N, 120°18′08″E	2.4	7.53	7.54	193.7	0.32–1.18	0.12–0.18	32.89–45.87	2.11–2.98	0.48–0.64	9.12–11.01	

For water and sediment chemical composition (i.e., N, P and organic matter), minimum and maximum ranges were given (n = 3). All samples were collected from 10:00 to 15:00.

**Table 2:** Repeated ANOVA detecting effects of different lakes and experimental period on turion sprouting and re-sprouting capacities and effects of different lakes, experimental period and scale leaf removal (treatment) on turion sprouting capacities (α = 0.05).

	d.f.	F	P
Sprouting experiment			
Lakes	1,52	76.4	<0.001
Time	2.7,141.8	208.7	<0.001
Lakes*Time	2.7,141.8	8.1	<0.001
Re-sprouting experiment			
Lakes	1,52	60.1	<0.001
Time	3.5,182.3	107.7	<0.001
Lakes*Time	3.5,182.3	5.2	0.001
Scale leaf removal experiment			
Lakes	1,48	46.4	<0.001
Treatment	2,48	0.4	0.688
Times	3.6,170.7	105.5	<0.001
Lakes*Treatment	2,48	1.5	0.227
Lakes*Times	3.6,170.7	6.3	<0.001
Treatment*Times	7.1,170.7	1.3	0.251
Lakes*Treatment*Times	7.1,170.67	0.2	0.981
